# The Potential Role of Ionic Liquid as a Multifunctional Dental Biomaterial

**DOI:** 10.3390/biomedicines11113093

**Published:** 2023-11-20

**Authors:** Md Iqbal Hossain, Abdullah Bin Shams, Shuvashis Das Gupta, Gary J. Blanchard, Ali Mobasheri, Ehsanul Hoque Apu

**Affiliations:** 1 Department of Chemistry, Michigan State University, East Lansing, MI 48824, USA; hossai13@chemistry.msu.edu (M.I.H.); blanchard@chemistry.msu.edu (G.J.B.); 2 The Edward S. Rogers Sr. Department of Electrical Computer Engineering, University of Toronto, Toronto, ON M5S 3G4, Canada; abdullahbinshams@gmail.com; 3 Research Unit of Health Science and Technology, Faculty of Medicine, University of Oulu, 90220 Oulu, Finland; shuvashis.dasgupta@oulu.fi (S.D.G.); ali.mobasheri@oulu.fi (A.M.); 4 Division of Public Health, Epidemiology and Health Economics, WHO Collaborating Center for Public Health Aspects of Musculo-Skeletal Health and Ageing, University of Liège, 4000 Liège, Belgium; 5 State Research Institute Centre for Innovative Medicine, 08410 Vilnius, Lithuania; 6 Department of Joint Surgery, The First Affiliated Hospital of Sun Yat-sen University, Guangzhou 510080, China; 7 Department of Biomedical Sciences, College of Dental Medicine, Lincoln Memorial University, Knoxville, TN 37923, USA; 8 Institute for Quantitative Health Science and Engineering, Department of Biomedical Engineering, Michigan State University, East Lansing, MI 48824, USA; 9 Division of Hematology and Oncology, Department of Internal Medicine, Michigan Medicine, University of Michigan, Ann Arbor, MI 48109, USA; 10 Centre for International Public Health and Environmental Research, Bangladesh (CIPHER,B), Dhaka 1207, Bangladesh

**Keywords:** biomaterials, dental materials, ionic liquids (ILs), tissue regeneration and multifunctional

## Abstract

In craniofacial research and routine dental clinical procedures, multifunctional materials with antimicrobial properties are in constant demand. Ionic liquids (ILs) are one such multifunctional intelligent material. Over the last three decades, ILs have been explored for different biomedical applications due to their unique physical and chemical properties, high task specificity, and sustainability. Their stable physical and chemical characteristics and extremely low vapor pressure make them suitable for various applications. Their unique properties, such as density, viscosity, and hydrophilicity/hydrophobicity, may provide higher performance as a potential dental material. ILs have functionalities for optimizing dental implants, infiltrate materials, oral hygiene maintenance products, and restorative materials. They also serve as sensors for dental chairside usage to detect oral cancer, periodontal lesions, breath-based sobriety, and dental hard tissue defects. With further optimization, ILs might also make vital contributions to craniofacial regeneration, oral hygiene maintenance, oral disease prevention, and antimicrobial materials. This review explores the different advantages and properties of ILs as possible dental material.

## 1. Introduction

The first ionic liquid (IL), ethylammonium nitrate, was reported in 1914 by Paul Walden [1]. In 1992, Wilkes and Zaworotko [2] reported the first 1-ethyl-3-methylimidazolium-based air and moisture-stable imidazolium-based ILs, garnering the attention of the research community. To date, this compound class has been used in most science and technology spheres. ILs are salts of relatively large organic cations and inorganic or organic anions. Generally, at least one of the ions is voluminous, asymmetric, and contains nonpolar tails or combinations of these properties. The bulky and asymmetric ions of ILs, compared to simple ions of classical inorganic salts, such as NaCl, prevent crystallization at ambient temperature, with melting points below 100 °C. When the melting points are near or below room temperature, the ILs are termed as room-temperature ionic liquids (RTILs) [3].

**Figure 1:**
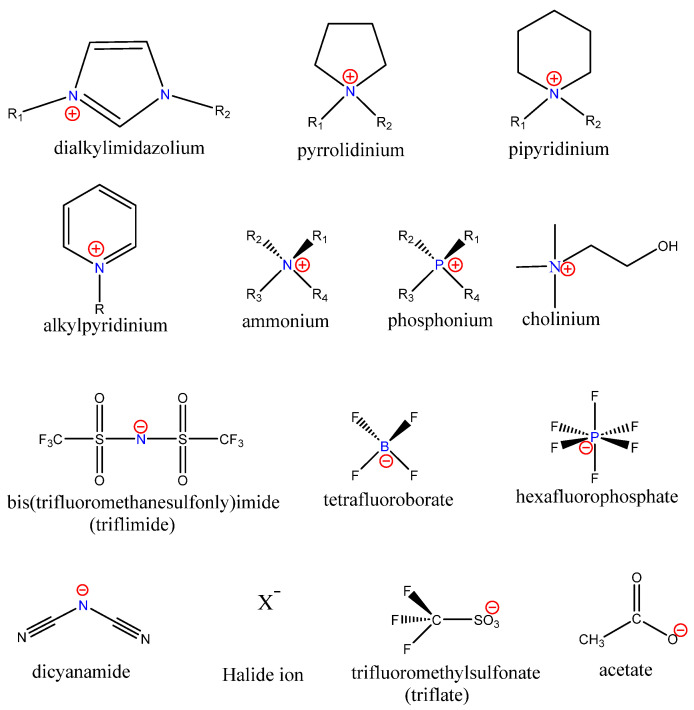
Chemical structures of some typical Ionic Liquid cations and anions [11].

ILs have drawn attention due to their attractive properties, including low vapor pressure, high chemical and thermal stability, wide electrochemical window, nonflammability, and the ability to dissolve various organic and inorganic materials. The properties of ILs, such as density, viscosity, and hydrophilicity/hydrophobicity, can be modified by judicious selection of cations and anions, by fine-tuning the lengths of the cation alkyl chain, or by the covalent tethering of task-specific functionalities to one or both of the constituent ions [4,5,6,7,8,9,10]. Hence, ILs are frequently referred to as task-specific or designer solvents. Cations and anions of some of the discussed ionic liquids are shown in Figure 1.

The unique properties of ILs make them particularly promising candidates as environmentally benign or “green” alternatives to organic solvents for chemical synthesis [12], catalysis [13], separation [14], and extraction [15]. Moreover, they represent safe and versatile electrolytes for electrochemical applications (lithium batteries, supercapacitors, and fuel cells) and photovoltaics (dye-sensitized solar cells (DSSCs) [16]). They are also novel functional materials [17] for lubrication [18], microfluidics [19], and sensors [20]. Additionally, the antimicrobial properties of ILs, discovered two decades ago, have facilitated their use in numerous biomedical applications as novel biomaterials.

Although few reviews have explained the potentiality of ILs in biomedical applications, none have discussed the potential role of ILs in dentistry and craniofacial sciences in details. This comprehensive review is a pioneering study in dentistry and craniofacial engineering, exploring the different functionalities and applications of ILs in this field.

## 2. Ionic Liquids as Antimicrobial Agents

According to the Centers for Disease Control and Prevention (CDC) of the United States (U.S.) [21,22], more than 3 million people become infected with antimicrobial-resistant pathogens, resulting in 48,000 deaths annually in the U.S. These infections are associated with a more than $4.6 billion annual burden on the health care system. These numbers will increase unless action is taken. Therefore, antimicrobial resistance is a pressing issue that must be overcome in global health. While overuse and misuse of antibiotics are growing concerns, the lack of novel antibiotics for resistant bacteria is the main challenge. Therefore, the search for next-generation antibiotics is a race against time. With high tunability and task-specificity, ILs have been envisaged as a promising next-generation antibiotic for resistant microorganisms [23].

**Figure 2:**
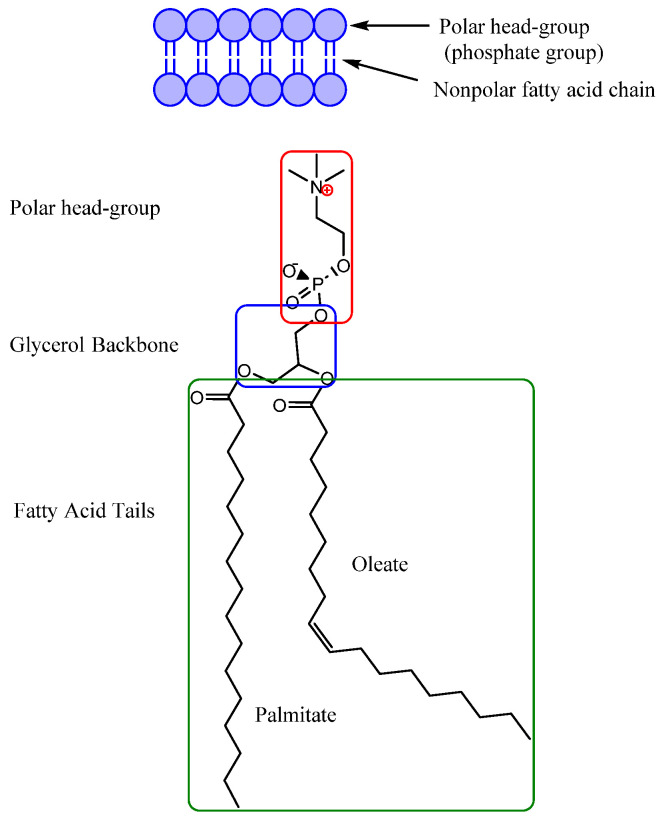
Different components of a model lipid bilayer (Phospholipid) [25].

Cell membranes, both plasma membranes or internal membranes are made of glycerophospholipids: a glycerol, phosphate group, and two fatty acid chains. Glycerol is a three-carbon poly alcohol that acts as the connector and attaches the phosphate polar (hydrophilic) head group and two nonpolar, hydrophobic hydrocarbon fatty acid tails [24]. The different components of a model lipid bilayer are shown in Figure 2. Cholesterols (not present in the bacterial cell membrane) regulate cell membrane fluidity (stiffness). Cholesterol also plays an essential role in maintaining the integrity of lipid membranes. Xiao-Lei et al. used an egg sphingomyelin (SM)-cholesterol model membrane to show that, in the absence of cholesterol, the meager molar IL: SM ratio can disrupt the model membrane [25]. Transmembrane proteins are embedded into the cell membrane and are responsible for the controlled transportation of materials into and out of the cell [26].

**Figure 3:**
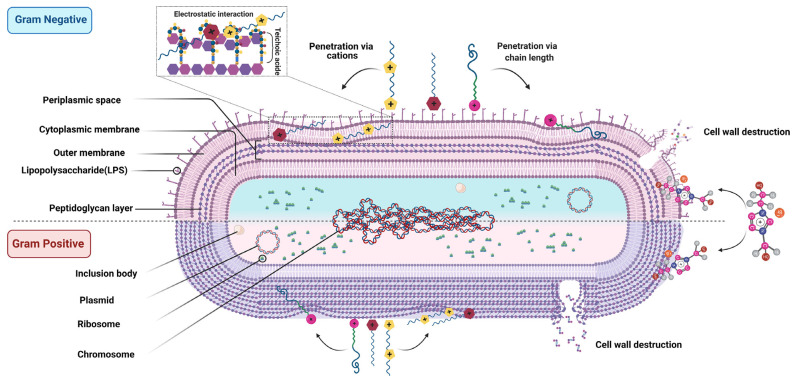
Illustration of how ionic liquids interact with the bacterial cell membrane (both Gram-positive and Gram-negative) and ultimately cause cell lysis. Reproduced with permission [34].

Due to the surface charge (phosphate head group) of the lipid bi-layer, the cationic moiety of the ILs adsorbed on the lipid membrane interacts with the transdermal protein and disorganizes the bi-layer with the penetration of its long alkyl chain, which ultimately changes the regular properties of a cell membrane [27,28,29]. The longer the alkyl chain, the higher the antibacterial activity [30]. Fluidity (stiffness) and membrane potential changes affect the biochemical gradients and interrupt the controlled exchange of intercellular and extracellular materials by affecting standard diffusion rates and transdermal protein stability. ILs sometimes initiate irreversible cell wall damage by creating permanent pores, another critical cause of the sub-cellular imbalance [31,32,33]. Overall, the antimicrobial activity of ILs can be divided into several steps. First, ILs are absorbed into cell membranes due to the electrostatic interactions of the polar head group. Second, they interact with the transmembrane protein. Third, they penetrate the phospholipid bilayer with their hydrophobic tails, disrupting the layer, causing intracellular cytoplasm leakage, and leading to cell lysis [34] (Figure 3). RTILs, based on imidazolium cation with a long alkyl chain and hydrophobic anion bis(trifluoromethansulfonyl)imide (NTf2), show antibacterial activity. The positive charge of the imidazolium ion is electrostatically attracted to the membrane’s negatively charged phosphate head groups while its alkyl long chain can easily penetrate the bacterial cell wall [35]. In particular, the hydrophobic anion (NTf2) of ILs may increase their antibacterial properties by disorganizing membranes.

### Parameters to Control Antimicrobial Properties

Antimicrobial properties can be enhanced by increasing the alkyl chain length. In a recent study, replacing an alkyl group with a phenolic group and increasing the other alkyl chain length of an imidazolium-based IL exhibited minimal inhibitory concentrations (MICs) < 7.81 µM against *Pseudomonas aeruginosa*, *Escherichia coli*, and *Staphylococcus aureus* [30]. ILs become absorbed into the cell membrane by electrostatic interactions between ILs and the polar head groups. Therefore, increasing the number of cations in per-ionic liquids can enhance the efficacy of ILs as antimicrobial agents [29,31]. Polymeric ionic liquids (PILs) are more effective than monomers as one can tune the hydrophobicity and charge density (number of cations) in PILs. Therefore, PILs with better efficacy and lower MIC values than their monomers are better antimicrobial agents for resistant bacteria [23].

## 3. Biomedical Applications of Ionic Liquids

A series of experimental and computational studies [36,37,38,39,40,41] have proven that when the ILs’ alkyl chain length becomes longer than four carbons, they aggregate and form a nonpolar domain. These nonpolar domains permeate the three-dimensional ionic networks. While the chain length increases, these nonpolar domains become larger and more connected, exhibiting microphase segregation. Ionic liquids show this property even in mixtures with water or other molecular solvents like DMSO, propylene carbonate, acetonitrile, and short-chain primary alcohol [9,10]. This unique property of ILs and their binary mixture with molecular solvents make them universal drug solubilizing agents. The limiting factor for different drug molecules is their poor solubility in biological media and cellular matrices mainly composed of water. RTILs are superior drug delivery materials compared to commonly used salts in pharmaceuticals since they do not possess crystal polymorphism problems and can dissolve organic and inorganic materials. Therefore, turning most drugs into ILs will enhance their therapeutic utility [42,43,44], making them an active pharmaceutical ingredient ionic liquid (API-IL). Targeted drug delivery encompasses drug packaging, transport to the targeted area, and controlled release. ILs can play a significant role in all areas as they circumvent biological barriers without hampering biological activity [45,46,47]. Different approaches, such as micelles, inverted micelles, vesicles, liposomes, nanoparticles, emulsions (micro or nano), hydrogels, etc., load drugs and carry them to the targeted area, then control release by photo or thermal sensitivity. ILs can be tailored easily to form emulsions (ILs-in-water and ILs-in-oil). ILs can be used as polar media in IL-in-oil or nonpolar media with a significant alkyl chain length in IL-in-water emulsions. By replacing one of the alkyl groups with task-specific functionality and a large alkyl chain in the other, ILs can form micelles without surfactants. Studies have found the highest drug loading and targeted drug delivery [48,49,50]. Properties of hydrogel, ionogels, and thermoresponsive gels can be tuned using task-specific ILs and can be used for controlled drug release [51,52].

The long-term stability of proteins is essential in terms of dealing with proteins. Generally, proteins are folded to avoid loss due to aggregation. Instead of aqueous buffers, ILs help prevent aggregation and reverse aggregation or refolding (only 3% loss) for many cycles [53,54,55]. Controlled manipulations of the polar and nonpolar regions of ILs and their binary mixture enable them to offer low toxicity and thermal and conformational stability [56]. Although it is unclear how ILs interact with proteins, combinations of experimental and theoretical approaches are needed.

## 4. Ionic Liquid-Based Applications in Craniofacial Engineering and Dentistry

### 4.1. ILs in Dental Implants

**Figure 4:**
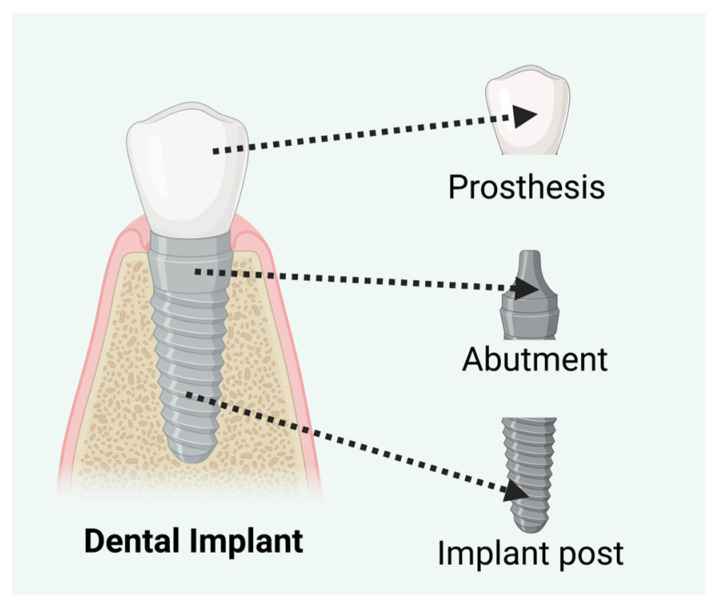
Components of a dental implant. Created with BioRender.com.

According to the American Academy of Implant Dentistry (AAID) and the American College of Prosthodontists, approximately 36 million U.S. citizens have lost all their teeth, and 120 million are missing at least one. These numbers are expected to grow over the next two decades. People can lose their teeth irrespective of age to numerous causes, including decay, gum disease, injury, accident, cancer, etc. Loss of a single tooth worsens overall oral health by weakening the jawbone. Dental implants are the most common treatment for dental loss. An implant consists of three components: an implant post, a cylindrical screw-shaped device anchored into the mandibular bone that provides necessary support for a dental prosthesis (crown), and an abutment that connects dentures to dental posts (Figure 4). Endo-osseous implants are made of metal, which must be biocompatible, non-corrosive, and flexible with high strength. Pure titanium and different titanium alloys are common materials for endo-osseous implants. Their long-term clinical survival rate has made them the gold standard. Zirconium ceramics have been introduced as an alternative to titanium due to drawbacks reported in the literature, such as allergies, hypersensitivity, metal degradation, and discoloration in the peri-implant regions [57,58,59].

**Figure 5:**
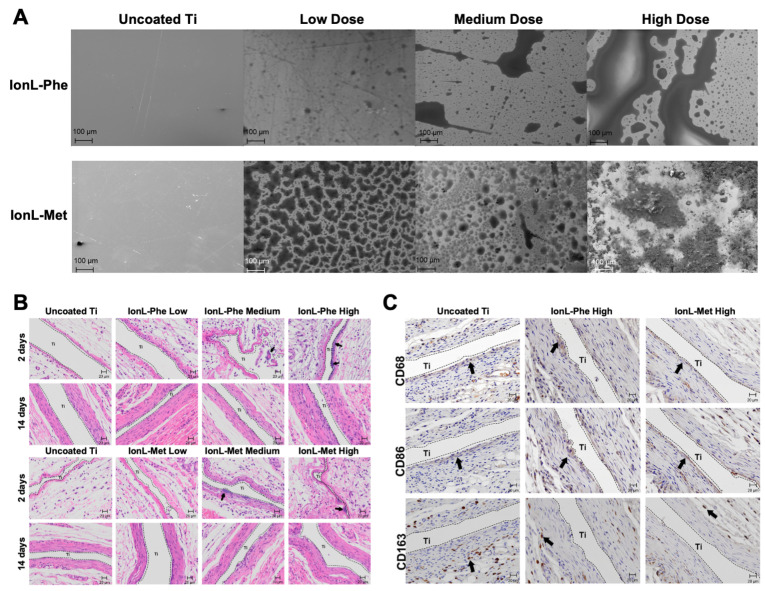
Ionic liquid-coated titanium implant. (**A**) SEM images of titanium surfaces coated and uncoated with ionic liquids at different doses (Scale bar 100 µm). (**B**) Hematoxylin and eosin staining of titanium implants (coated and uncoated). Images show the healing representation (pointed with arrows) of post-implantation at 2 and 14 days (Scale bar 20 µm). (**C**) Areas marked with arrows showing inflammatory responses of surrounding tissues at 14 days (post-implantation) in coated and uncoated titanium implants (Scale bar 20 µm). Reproduced with permission [71].

A successful dental implant is associated with the osseointegration of surrounding soft and bone tissues with the dental implant surface [60,61,62,63]. However, many factors, such as microbial biofilm formation, stress during insertion and mastication of the implant, corrosion, and systematic health and host immune-inflammatory responses, may affect the implant’s long-term stability [62]. Therefore, implants are subjected to different processes, including sandblasting, selective ion etching, and bioactive coating to minimize surface roughness, formation of an anti-bacterial biofilm, etc. [64]. Implant surfaces are prone to bacterial colonization, resulting in a “race for the surface” between host tissues and bacteria. Therefore, anti-bacterial biofilm is a determining factor for the long-term stability of the dental implant and is considered a major determining factor of implant failure [65,66]. Zhao et al. have shown how the “race for the surface” occurs using human gingival fibroblasts and different supragingival bacterial strains [66]. Within the first hour, a protective soft tissue seal must form around the implant’s neck to prevent bacterial colonization. If this does not occur (i.e., bacteria form a biofilm), fibroblasts lose the battle, and the implants must be replaced. ILs, especially dicationic ILs, have been used to prepare anti-bacterial biofilms, providing essential lubrication, corrosion resistance, and wear performance while maintaining compatibility with the host cells [67,68]. Since ILs act as antimicrobial agents, creating an IL layer can help provide conditions for fibroblast and pre-osteoblast growth and proliferation to form a seal, preventing bacterial growth [67,69,70]. Wheelis et al. evaluated the biocompatibility of dicationic IL coatings on commercially pure titanium disk (cpTi) in a subcutaneous implantation rat model [71]. They used two dicationic ionic liquids with two amino acid anions (IonL-Phe, IonL-Met) and three doses to investigate the interference of coatings in the osteointegration process during the early healing period (Figure 5). They did not observe significant interference in the early healing timeline or tissue regeneration. They also showed in separate studies [67,70] that dicationic IL film remains on the implant surface for more than seven days, maintaining growth conditions for human gingival fibroblasts and pre-osteoblasts while posing severe toxicity to bacterial cells, thus helping the host cells to commandeer the surface.

### 4.2. ILs as Dental Infiltrant Materials

Non-cavitated carious lesions near dental surfaces are a prevalent dental disease [72]. To protect dental hard tissues, restorative materials are placed directly into a tooth cavity to prevent further expansion of lesions and restore dental functions and aesthetics. The American Dental Association (ADA) classified restorative materials into four categories in 2003: amalgam, resin-based composites, glass ionomer, and resin-modified glass ionomer. Proper diet, patient education on oral hygiene, and topical fluoride application are the primary treatments for incipient enamel caries lesions [73,74]. The next step is to apply biocompatible materials as resin infiltrates for superficial carious lesions. These infiltrates are light-curable resins that inhibit carious progression by sealing the lesion’s body and pores in proximal dental surfaces [75,76,77].

**Figure 6:**
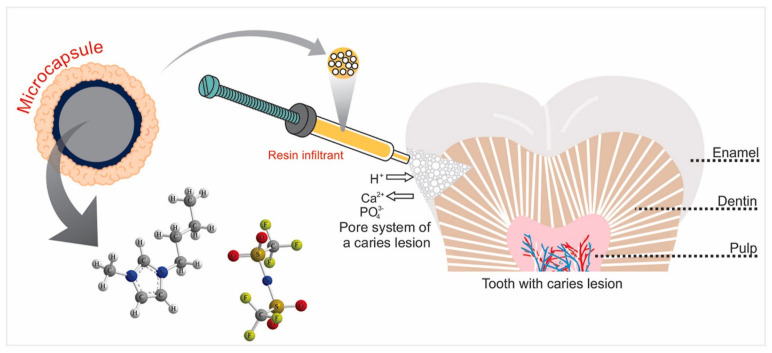
Microcapsules loaded with ionic liquids as dental resin infiltrates. Reproduced with permission [78].

Designing highly active antimicrobial surfaces or coatings is a significant challenge for public health. Resins are inert methacrylate monomers, such as triethylene glycol dimethacrylate (TEGDMA) and bisphenol A-glycidyl methacrylate (Bis-GMA), that lack antimicrobial activity. Hence, efforts are being made to endow resin infiltrates with antimicrobial properties. Cuppini et al. developed microcapsules loaded with 2.5wt%, 5wt%, or 10wt% ionic liquid as resin infiltrates [78] (Figure 6).

Microcapsule-loaded RTILs show excellent antibacterial properties without changing other physicochemical properties or eliciting cytotoxicity (cell viability > 90%). In recent studies [79,80], three thermally stable epoxy-amine networks were synthesized using imidazolium-based ionic liquid monomers with similar thermomechanical properties to conventional diglycidyl ether (DGEBA) epoxy prepolymers. They further tested antimicrobial properties against *Escherichia coli* (*E. coli*) and found potent biofilm inhibition of ~92%. Importantly, imidazolium ionic liquids can replace carcinogenic bisphenol A. Recently, an RTIL (BMIMTFSI) was successfully used as an antibacterial experimental orthodontic adhesive against *Streptococcus mutans* [81] and resin infiltrate [78].

Nanoparticles, especially silver (AgNP), zinc oxide, titanium dioxide nanoparticles, quaternary ammonium, and cationic nanoparticles, have been incorporated into resin as antibacterial agents, showing promising results [82,83,84]. Each of these methods has challenges. For example, the primary concern of AgNPs is adverse effects on human health and the dispersion of nanoparticles into solvents. RTILs are used to stabilize nanoparticles and act as antimicrobial agents for dental resins [81,85,86]. Although microorganisms can rapidly develop resistance against nanomaterials, such as AgNP, the infinite tunability of RTILs makes them the ultimate solution for antimicrobial resistance [35,87].

### 4.3. Ionic Liquids (ILs) as Oral Hygiene Products

Efforts have been made to develop IL-based oral hygiene products, such as dental toothpaste and mouthwash/mouth rinse. Madhusudan et al. proposed an oral care composition for removing or reducing plaque comprising an IL formulated with choline salicylate and tris-(2-hydroxyethyl) methylammonium methylsulfate. They developed 74 prototype formulations; in vivo experimental results suggested several formulations might provide clinical efficacy to disrupt, dissolve, and remove bacterial plaque. The function of ionic liquid-based oral care compositions will also prevent different gingivitis and oral cavity diseases, such as dental caries, calculus, erosion, periodontitis, halitosis, and even salivary gland disorders, including xerostomia/dry mouth [88].

### 4.4. Ionic Liquids (ILs) as Dental Restorative Materials in Clinics

In clinics, dental cement selection is vital in achieving successful restoration and significantly increases the duration of teeth restoration [89]. Kajimoto et al. explored the possibility of utilizing ILs to mix with dental cement, converting them to multifunctional smart (intelligent) adhesives [90]. Due to the bonding strength of the dental cement, removal from the oral cavity can require excessive force or vibration to the tooth by electrical appliances, which might generate heat. Heating adhesives in the oral cavity have possible drawbacks and risks damaging the surrounding oral mucosa [90]. The group experimented with a specific electric current to trigger and control the heating process by implementing IL-based dental cement. They developed a prototype resin-modified glass-ionomer cement (RMGIC) with an IL and validated the use of an electric current trigger to control the properties of an IL-based smart cement [90]. In a more recent study, the same research team reported that after immersion and decreasing the bonding strength via electrical current application, the electrical conductivity was greater for the RMGIC with IL [91]. Thus, combining dental cement and ILs might represent an effective multifunctional smart material with antimicrobial properties.

## 5. Biosensor Prospects in Dental Applications

### 5.1. Interleukin-6 Sensor to Detect Oral Cancer

Researchers have shown that interleukin-6 (IL-6) promotes tumor growth by causing DNA methylation changes, which can lead to changes in the oral cancer cell gene expression [92]. Higher levels of IL-6 have been reported in the serum and saliva of patients with oral cancer than in control subjects, demonstrating a possible relationship between IL-6 and oral cancer [93].

In the future, oral health care could utilize ionic liquids (ILs) as a strategy for early oral cancer diagnosis. IL with menthol can be used to mass-screen patients for early oral cancer diagnosis. The IL’s chemical structure can affect the menthol release rate [94]. Complexes can be formed comprising IL with a menthol moiety [94] and IL-6 antibodies [95]. This complex can bind IL-6 and release a proportional amount of menthol. As menthol triggers TRPM8 and induces a cooling sensation, the intensity of this sensation will notify the patient if IL-6 has been released and the amount of IL-6 in the mouth.

### 5.2. Gingivitis Sensor

A common condition in many oral diseases, such as gingivitis, is inflammation. Microorganisms cause an inflammatory immune response mediated by changes in the vascular network and the exudation of gingival crevicular fluid (GCF), which contains inflammatory and plasma cells, ultimately resulting in tissue death. Gingivitis is the primary cause of bleeding gums that allows plaque to accumulate at the gum line. Tartar will form if plaque is left untreated. This results in bleeding as well as periodontitis, a severe form of gum and jawbone disease [96]. Functionalized IL can be used for early diagnosis of gingivitis and to highlight the damaged gum area. A possible option would be to synthesize fluorescent IL with light intensity that becomes amplified or quenched upon illumination in the presence of hemoglobin [97]. Hemoglobin is the oxygen-carrying protein in red blood cells [98]. Therefore, after rinsing the mouth with the IL complex, fluorescent light would reveal the bleeding sites in the gum. An alternative option would be to make menthol-based IL functionalized for hemoglobin. In this case, the IL can be synthesized to release menthol when in contact with hemoglobin, causing a cooling sensation in the areas affected by gingivitis.

### 5.3. Tooth Caries and Cracks Sensor

The most prevalent cause of cracked tooth syndrome is chewing on hard foods, which causes an incomplete tooth fracture. This is the third most frequent cause of tooth loss [99], with no suitable diagnosis method. Transillumination and radiographic approaches, traditional dental crack identification methods, are inaccurate and offer subpar imaging resolution [100]. Light-induced fluorescence dental imaging Field [100,101,102] has recently gained considerable attention as a useful tool for diagnosing small cracks and cracked teeth at an early stage. For real-time diagnosis, fluorescent IL can be used [103]. Here, after rinsing the mouth with the liquid complex, the dyes will fill all possible cracks and, upon illumination, will reveal the location and size of the cracks. It is important to note that the illumination angle is essential to correctly identify the position and depth of the cracks [101]. This method provides a low-cost visual diagnostic tool for occlusal dental caries, proximal dental caries, and tooth cracks. With the recently emerging telemedicine, this technique will make clinical practice more accessible, especially in rural areas of developing countries.

### 5.4. Breath-Based Sobriety Sensors

Drinking too much alcohol can result in overdose, car accidents, intoxication of athletes/pilots, etc. According to studies, the blood and saliva alcohol levels are consistent [104]. A driver’s sobriety can be evaluated using a saliva alcohol test. The main component of alcoholic drinks is ethanol, and saliva typically lacks alcohol subtypes, such as methyl, propyl, or allyl. Ethanol concentration can be identified using ILs [105]. This can be paired with the colorimetric IL to form an ethanol sensor. This hybrid liquid can be used to rinse the mouth for a few seconds. Depending upon the alcohol concentration in the saliva, the color of the liquid would change accordingly and provide a visual indicator of the blood alcohol level. This method can be used as a fast, low-cost, and scalable technique to monitor sobriety.

### 5.5. Oral Hygiene Sensor

A person’s oral cavity contains more than 70 different species of bacteria. Although most are harmless, some bacteria are dangerous and can lead to dental problems, including tooth decay and gum disease [106]. Oral health can be improved, and the risk of dental problems can be decreased by minimizing the number of harmful germs in the mouth. Fluorescent IL (excitation in the visible spectrum) functionalized for harmful bacteria can be synthesized as a toothpaste [107]. This can be used by individuals at home to monitor the effectiveness of brushing based on the number of fluorescent areas. This will significantly help to improve personal hygiene.

## 6. Toxic Effects of Ionic Liquids (ILs)

Ionic liquids (ILs) have long been considered environmentally friendly and ecologically benign solvents. Nevertheless, concerns have recently arisen regarding their potential toxicity to human health and environmental impact, necessitating a thorough investigation. ILs are engineered to possess low volatility and stability, which, unfortunately, reduces their biodegradability [108]. The toxicity of ILs is contingent upon their chemical composition, including the types of cations, anions, and the alkyl chain length. ILs were found to be toxic or highly toxic toward cells and living organisms [109,110]. Besides hampering the growth rate of microbes, their antibacterial activity also interferes with their productivity [111].

Notably, ILs featuring choline-based components exhibit lower toxicity than the commonly used imidazolium, pyridinium, and pyrrolidinium variants [112,113,114,115]. This underscores a clear relationship between IL structure and toxicity, offering prospects for developing less harmful ILs. Even appropriate anions can reduce the half-maximal effective concentration (EC50) values by three orders of magnitude [116]. It is worth noting that when ILs encounter water or other polar solvents, they may undergo ion exchange, forming new substances with potentially different toxic properties. Research endeavors have been undertaken to assess the cytotoxic effects of various piperidinium and pyrrolidinium ILs on the MCF7 human breast cancer cell line. Results suggest that toxicity tends to increase with longer alkyl chain lengths. Furthermore, the nature of the anions also plays a role, with Tf2N appearing to be more toxic than Br- [117]. Similar findings have been observed in studies involving *Escherichia coli*, *Staphylococcus aureus*, *Bacillus subtilis*, *Pseudomonas flurescens,* and *Saccharomyces cerevisiae*, reinforcing the trend that higher alkyl chain lengths are associated with greater toxicity [118,119]. However, further studies are needed to establish the precise molecular mechanism underlying the toxic effects of ILs.

With proper optimization, ILs can be incorporated into drug formulations to avoid the development of cancer treatment resistance. Adequate knowledge of the toxicity will provide vital information for anti-cancer drug development, as apoptosis-related drug resistance is challenging for different cancer types. Additionally, there is an interest in identifying other means of inducing cytotoxicity [120]. This information will improve the efficacy of drugs against cancer progression and neurodegenerative disease.

## 7. Prospects and Future Directions

Room temperature ionic liquids (RTILs) have high potential in advanced tissue engineering applications, including craniofacial engineering and dental procedures. They can be easily incorporated with self-healing materials [121], magnetoelectric nanoparticles (MENPs) [122,123], and electroconductive materials [124], through advanced tissue engineering applications, such as three-dimensional (3D) cell cultures [125] and 3D bioprinting techniques [126]. They can also be easily combined or used to control multi-functional material-based applications. For example, they can influence applications based on MENPs and electroconductive materials [122], and can be used for implant sensing [127].

### 7.1. ILs with Electroconductive Material for Dental Tissue Regeneration

Most cell types respond to electrical stimuli, which control the healing and regeneration of skin wounds, spinal cord injury, bone fractures, and neural growth [128]. The combined usage of electroconductive materials and ILs has huge potentiality for guided cell and tissue regeneration and may enable new directions for guided oral tissue regeneration [124]. One of the challenges of dental hard tissue regeneration is the variable growth rate, resulting in its under- or over-growth. The ILs can easily solve this issue by providing better tissue regeneration control. ILs play a vital role in neural degeneration [129] and could be an ideal root canal material to revitalize the dental pulps. By combining multifunctional magnetoelectric nanoparticles (MENPs) with electroconductive and biodegradable materials, ILs will have a potential role in the formulation of smart dental materials [130].

### 7.2. A Critical Component for Preventive Dental Care and Oral Hygiene

Oral hygiene maintenance is critical for a healthy oral cavity and lifestyle. However, unfortunately, due to musculoskeletal disorders such as osteoarthritis, rheumatoid arthritis, and other conditions, many elders find it challenging to maintain good oral hygiene [131]. Also, those who have arthritis cannot correctly grip a toothbrush [131,132], representing a primary concern for poor oral hygiene, which leads to microbial accumulation and dental plaque formation. Also, Niesten et al. reported that elders residing in institutions generally discontinue oral hygiene maintenance mainly due to inadequate social support and disorientation [132]. Therefore, a need exists to provide improved methods and oral care products, such as toothpaste and mouthwash, for plaque removal/prevention to overcome some of these inefficiencies arising from poor brushing and flossing techniques. In the future, ILs can minimize the gap by improving the antimicrobial properties of toothpaste and mouthwash. This could effectively remove plaque usually unnoticed between teeth, in the teeth cavities and fissures, and in gum pockets. Madhusudan and colleagues with Colgate-Palmolive Company (New York, NY, USA) invented such a concept to integrate ILs in oral care compositions [88].

With advanced 3D bioprinting techniques, there are enormous opportunities for combining various amounts of ILs in toothpaste/mouthwash compositions to enhance the protective environment within the oral cavities. Hayashi et al. recently explored a technique enabling ILs to formulate fluoridated toothpaste. They reported preparing a new class of hybrid ILs, called “fluoride ion-encapsulated germoxane cages”, containing a fluoride ion inside [133].

Breath-based sensors could be another area for future bench-side applications of ILs in clinical dentistry. There is evidence that the presence of chronic disease and cancer alters cellular metabolic processes, and these alterations are recorded in the released volatile organic compound (VOC) compositions of cancer cells. We have shown the potential of using VOC sensors as a biomedical engineering approach for oral cancer detection. Due to their multifunctional nature, ILs could be used for such breath-based early detection systems [134].

### 7.3. As a Routine Anti-Microbial Material for Dental Clinics

Like any healthcare facility, dental clinics pose a risk of spreading coronavirus disease (COVID-19) via cross-infection among patients, dentists, and community members. In clinical dental settings, the instruments produce aerosols, droplets of saliva, secretions, saliva, and blood, which can rapidly transmit viruses between dental practitioners, assistants, and patients and their attendants [135,136]. Due to their antimicrobial properties and chemical characteristics, RTILs could be used for coating the walls, appliances, and surfaces in dental clinics to provide an infection-free environment [137].

A denture appliance resides in the oral cavity and is highly prone to microbial growth, bacterial biofilm formation, and contamination. Many antimicrobial agents are commonly used for denture preparation, such as titanium dioxide, methacrylic acid monomers, silica, and MPDB/12-methacryloyloxy dodecylpyridinium bromide. Due to their multifunctional properties and antimicrobial nature, RTILs can be incorporated as routine agents to fabricate anti-viral dentures [137].

Further studies are required to accurately assess the toxicity at the cellular and molecular levels. Experiments should be conducted utilizing flat two-dimensional (2D) cultures and more advanced three-dimensional (3D) in vitro models (i.e., spheroids, sandwich cultures) with cells embedded in extracellular matrices, such as rat tail-derived collagen type 1 and mice sarcoma tissue-derived basement membrane mimicking matrices [125,138]. After successfully conducting biological characterization with in vitro models, in vivo studies are required to complete the picture of IL toxicity prior to biomedical applications.

## 8. Conclusions

Ionic liquids (ILs) have a unique multifunctional property that might allow researchers to explore the potentiality of remote sensing and controlling cellular growth in the craniofacial region. ILs are antimicrobial and easy to characterize, enhancing the functionality of dental cement, 3D bioprinting, and implants with sensing capabilities. However, further in vitro and in vivo studies are required to evaluate the cytotoxicity of ILs and optimized their formulations.

## Data Availability

Data are contained within the article.
